# Dynamic of Serum TWEAK Levels in Critically Ill COVID-19 Male Patients

**DOI:** 10.3390/jcm11133699

**Published:** 2022-06-27

**Authors:** Marijana Mikacic, Marko Kumric, Martina Baricevic, Daria Tokic, Sanda Stojanovic Stipic, Ivan Cvitkovic, Daniela Supe Domic, Tina Ticinovic Kurir, Josko Bozic

**Affiliations:** 1 Intensive Care Unit of the Department of Internal Medicine, University Hospital of Split, 21000 Split, Croatia; mmikacic@kbsplit.hr (M.M.); maolah@kbsplit.hr (M.B.); 2 Department of Pathophysiology, University of Split School of Medicine, 21000 Split, Croatia; marko.kumric@mefst.hr (M.K.); ivan.cvitkovic93@gmail.com (I.C.); tticinov@mefst.hr (T.T.K.); 3 Department of Anesthesiology and Intensive Care, University Hospital of Split, 21000 Split, Croatia; dariatokic@gmail.com (D.T.); sandastojanovicstipic@gmail.com (S.S.S.); 4 Department of Health Studies, University of Split, 21000 Split, Croatia; daniela.supe.domic@ozs.unist.hr; 5 Department of Medical Laboratory Diagnostics, University Hospital of Split, 21000 Split, Croatia; 6 Department of Endocrinology, Diabetes and Metabolic Diseases, University Hospital of Split, 21000 Split, Croatia

**Keywords:** TWEAK, biomarker, COVID-19, inflammation, acute respiratory distress syndrome, testosterone

## Abstract

Although the number of cases and mortality of COVID-19 are seemingly declining, clinicians endeavor to establish indicators and predictors of such responses in order to optimize treatment regimens for future outbreaks of SARS-CoV-2 or similar viruses. Considering the importance of aberrant immune response in severe COVID-19, in the present study, we aimed to explore the dynamic of serum TNF-like weak inducer of apoptosis (TWEAK) levels in critically-ill COVID-19 patients and establish whether these levels may predict in-hospital mortality and if TWEAK is associated with impairment of testosterone levels observed in this population. The present single-center cohort study involved 66 men between the ages of 18 and 65 who were suffering from a severe type of COVID-19. Serum TWEAK was rising during the first week after admission to intensive care unit (ICU), whereas decline to baseline values was observed in the second week post-ICU admission (*p* = 0.032) but not in patients who died in hospital. Receiver-operator characteristics analysis demonstrated that serum TWEAK at admission to ICU is a significant predictor of in-hospital mortality (AUC = 0.689, *p* = 0.019). Finally, a negative correlation was found between serum TWEAK at admission and testosterone levels (r = −0.310, *p* = 0.036). In summary, serum TWEAK predicts in-hospital mortality in severe COVID-19. In addition, inflammatory pathways including TWEAK seem to be implicated in pathophysiology of reproductive hormone axis disturbance in severe form of COVID-19.

## 1. Introduction

Ever since it emerged in late 2019, coronavirus disease 2019 (COVID-19) has rapidly developed into the biggest global healthcare issue [1]. The reasons behind very poor outcomes at the beginning of pandemic were multifactorial, yet what substantially contributed was poor understanding of the pathophysiological pathways encompassing the COVID-19 infection, especially with regards to aberrant immune response [2,3]. In fact, clinical experience with the use of both immunomodulatory agents and research data implies that immune response is a major culprit in severe respiratory insufficiency seen in COVID-19 [4,5,6,7]. Although the pandemic now seems to fade, clinicians endeavor to establish indicators and predictors of such responses in order to optimize treatment regimens for future outbreaks of SARS-CoV-2 and similar viruses.

Specifically, it has been suggested that human coronaviruses, including COVID-19, frequently lead to death via development of cytokine storm syndrome and consequent acute respiratory distress [8,9]. Multiple data support these findings. For instance, serum levels of pro-inflammatory cytokines, such as interleukin-6 (IL-6), tumor necrosis factor-alpha (TNFα), and IL-10, are elevated in patients with COVID-19, especially in those treated in the intensive care unit, correlating positively with disease severity [10,11,12].

TNF-like weak inducer of apoptosis (TWEAK), a member of the TNF ligand family, is a multifunctional cytokine extensively distributed in cell types and tissues [13,14]. By binding to its only known receptor, factor-inducible 14 (Fn14), TWEAK stimulates the release of the above-noted pro-inflammatory cytokines [15]. Although initially described as an apoptosis inducer, accumulating evidence has demonstrated that binding of TWEAK to the Fn14 contributes to the pathophysiology of diverse pathologic processes such as angiogenesis, carcinogenesis, and inflammation [16,17,18,19]. Recently, it has been suggested that TWEAK is closely associated with signaling pathways that cause alterations of intracellular signaling cascades in the lungs and thus contributes to the development of respiratory diseases of various etiologies (e.g., pulmonary artery hypertension, asthma, idiopathic pulmonary fibrosis) [20]. Notably, regardless of the underlying etiology, inhibition of TWEAK/Fn14 axis seem to contribute to attenuation of disease progression, thus making it an interesting therapeutic target for the aforementioned pathologies.

Studies suggest that disturbance of hypothalamic–pituitary–gonadal (HPG) axis is a two-way street in COVID-19. Specifically, although testosterone is known to attenuate oxidative stress and endothelial dysfunction, thus dampening the aberrant immune response, high testosterone levels could also lead to increased susceptibility of SARS-CoV-2 and portend worse outcomes by up-regulating the expression of transmembrane protease serine 2 (TMPRSS2) [21].

In the present study, we aimed to explore the dynamic of serum TWEAK levels in critically ill COVID-19 male patients and establish whether these levels may predict in-hospital mortality. Furthermore, we explored the association between circulating levels of TWEAK and testosterone levels. Finally, we aimed to establish a correlation between serum TWEAK and indices of myocardial injury and lung involvement in a severe form of COVID-19.

## 2. Materials and Methods

### 2.1. Study Design and Ethical Considerations

The present single-center cohort study was conducted at the University Hospital of Split Respiratory Intensivist Center (RIC) from December 2021 to May 2022. The study was approved by the Ethics Committee of the University Hospital of Split and was conducted in accordance with all ethical principles of the 2013 Declaration of Helsinki. Prior to enrollment in the study, all participants were acquainted with the details of the study and personally signed the informed consent.

### 2.2. Participants and Inclusion/Exclusion Criteria

The study involved 66 men between the ages of 18 and 65 who were suffering from a severe type of COVID-19 at the RIC of the University Hospital of Split. All included patients were intubated and placed on mechanical ventilation on the first day of admission to RIC. In the time of participant enrollment, the dominant variant of SARS-CoV-2 in Croatia was the SARS-CoV-2 B.1.617.2 (Delta) variant [22]. The standard references for endotracheal intubation included the following: airway protection, severe decompensate acidosis (pH < 7.2), and severe absolute hypoxemia (PaO_2_ < 50 mmHg or SpO_2_ < 90%) despite maximal noninvasive respiratory support. Exclusion criteria were: previously diagnosed malignant disease, previously diagnosed autoimmune disease, heart failure, renal failure, liver failure, vitamin D supplementation, and uncooperative patients. The study included male patients exclusively, as the principal aim was to establish the association between relevant inflammatory parameters, testosterone hormonal axis, and disease severity/outcomes in patient with severe COVID-19.

### 2.3. Clinical and Laboratory Evaluations

Demographic characteristics and relevant data from past medical history (comorbidities, chronic therapy, current therapy, time from disease onset to hospital admission, time from admission to ICU) were collected from the hospital records for each included patient. Vital signs and SpO_2_ were continuously monitored upon admission to the RIC. Body weight (kg) and height (cm) measurements were obtained from the medical record, and BMI was calculated by the body weight (kg) being divided by height-squared (m^2^).

Arterial blood gas variables and standard laboratory panel (complete blood count, white blood cells (WBCs), C-reactive protein (CRP), lactate dehydrogenase (LDH), and D-dimer) were measured upon admission and henceforth on a daily basis. All laboratory analyses were conducted using standard operating procedures at the Department for Laboratory medicine of the University Hospital of Split.

For risk stratification, we used multiple risk scores. The Brixia scoring system, an 18-point chest X-ray (CXR) scoring system, was used for grading of CXR reports into five different severity categories in hospitalized patients with COVID-19 infection [23]. Brixia score was calculated based on chest X-ray that was performed as a standard of care at admission to RIC. Simplified Acute Physiology Score (SAPS II), a score that consists of 12 physiological variables and 3 disease-related variables, was used to estimate in-hospital mortality [24]. Horowitz index, calculated by the ratio of SpO_2_ and FiO_2_, was used to assess lung function [25]. Both SAPS-II and the Horowitz index were calculated upon admission to the RIC. Survival of Severely Ill COVID (SOSIC) scores and SOSIC-1, -7, and -14 were used to predict the likelihood of 90-day mortality post-ICU admission [26], and calculations were performed at admission to RIC, 7 days after that, and 14 days after admission.

Peripheral venous blood samples for TWEAK measurement were collected at 3 points in time: upon admission to RIC, 7 days after, and 14 days after RIC admission unless patient was discharged or deceased. The blood samples were centrifuged at 3000× *g* for 10 min, aliquoted, and subsequently stored at −80 °C. On the evaluation day, serum was melted at room temperature. The serum concentrations of TWEAK (Phoenix Pharmaceuticals Inc., Burlingame, CA, USA) were determined using commercially available ELISA kits. All assays were conducted by an experienced biochemist according to the instructions of the manufacturers. Inter-assay and intra-assay reliability of the TWEAK ELISA were CV < 15% and <10%, respectively. The minimum detectable concentration of TWEAK was 62.5 pg/mL.

The following standard therapeutic methods and up-to-date protocols were used in patient treatment: corticosteroids, antiviral medications, anticoagulants, oxygen, and other supportive therapies. All patients were followed until discharge from hospital or death event during in-hospital stay, including both time spent in the RIC and time spent in other hospital departments.

### 2.4. Statistical Analysis

Data were analyzed by using software MedCalc (MedCalc Software, Ostend, Belgium, version 17.4.1) and Prism 6 for Windows^®^ (version 6.01, GraphPad, La Jolla, CA, USA). Categorical data are shown as absolute numbers (N) and percentages (%), while continuous data are shown as mean ± standard deviation (SD) or median (interquartile range). The normality of data was assessed with the Kolmogorov–Smirnov test. Chi-square (χ^2^) test for or Fisher’s exact test were used for the analysis of categorical variables as appropriate. Baseline data between principal groups of interest (patients who survived until discharge vs. patients who deceased during in-hospital stay) were compared either with Student’s *t*-test or Mann–Whitney U test as appropriate. Furthermore, the accuracy of TWEAK in predicting in-hospital mortality was tested using ROC analysis with a calculation of area under the curve (AUC). To compare whether there was statistically significant difference in TWEAK levels at different time points since admission, Friedman test with post hoc Conover test was employed. Finally, to examine correlation between serum levels of TWEAK and hs-TnI levels, CRP, Brixia score, SAPS-II score, SOSIC scores, Horowitz index, TSH levels, and vitamin D, we employed Spearman’s rank-order correlation analysis. In this analysis, the r correlation coefficient (rho), two-tailed significance (*p*) values, and respective correlation graphs were generated. The *p*-value < 0.05 was considered statistically significant for all analyses.

## 3. Results

The study population consisted of older adult male patients (average age of 55.1 years). A mortality event occurred in 13 of the 66 individuals who were enrolled (19.7%). Patients who died during in-hospital stay were older (*p* = 0.047), and more of them acquired nosocomial infection (10 out of 13 vs. 18 out of 53, *p* < 0.001). These patients also had greater prevalence of diabetes and hypertension (*p* < 0.001 and *p* = 0.004, respectively) and lower levels of vitamin D. In addition, LDH levels and high-sensitivity troponin I (hs-TnI) levels were lower in a subgroup of patients who survived until discharge, *p* = 0.024 and *p* = 0.002, respectively. Other clinical features of interest, comorbidities, and laboratory parameters showed no statistically significant differences (Table 1).

A panel of clinical scores for severity of the disease and/or prognosis were calculated and are presented in Table 2. All of the three SOSIC scores were higher in group that suffered from fatal event (*p* = 0.013, *p* < 0.001, and *p* < 0.001, respectively). In addition, Brixia score was also higher in group of patients who died during in-hospital stay (*p* = 0.004). Other risk scores did not differ between patients who died as a result of a fatal event during in-hospital stay and those who did not.

Serum levels of TWEAK rose during the first week after admission to ICU, whereas decline to baseline values was observed in the second week post-ICU admission (181.85 pg/mL (95.90–348.17) vs. 197.77 pg/mL (136.03–471.52) vs. 188.55 pg/mL (139.71–377.51), *p* = 0.032). On the other hand, in the subgroup of patients who died in hospital, serum TWEAK levels rose in the first 7 days but failed to decline in the subsequent week (247.2 pg/mL (89.7–326.8) vs. 284.20 pg/mL (157.64–582.86) vs. 293.80 pg/mL (188.55–640.51), *p* = 0.016).

**Figure 1:**
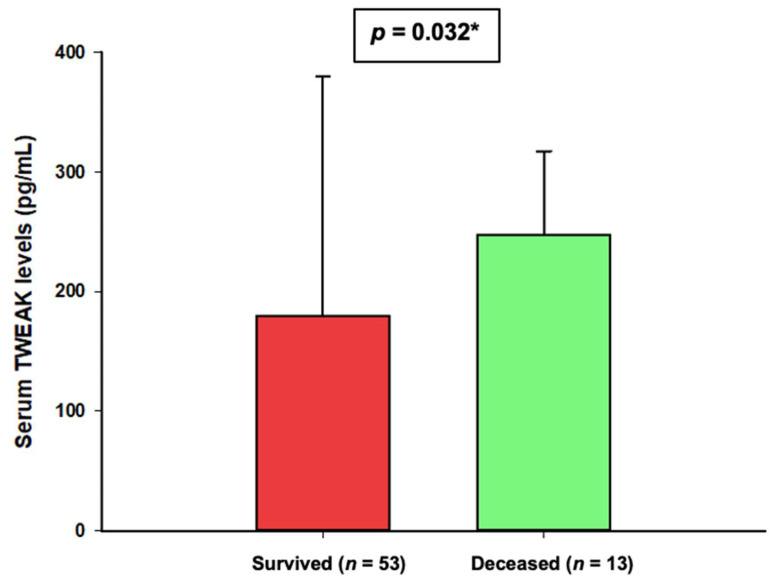
Comparison of serum TWEAK with regards to in-hospital mortality. Data are presented as median and 75th percentile * Mann–Whitney U test.

Serum levels of TWEAK at admission to ICU were lower in the subgroup of patients who survived (179.3 pg/mL (86.6–375.5) vs. 247.2 pg/mL (89.7–326.8), *p* = 0.037) (Figure 1).

**Figure 2:**
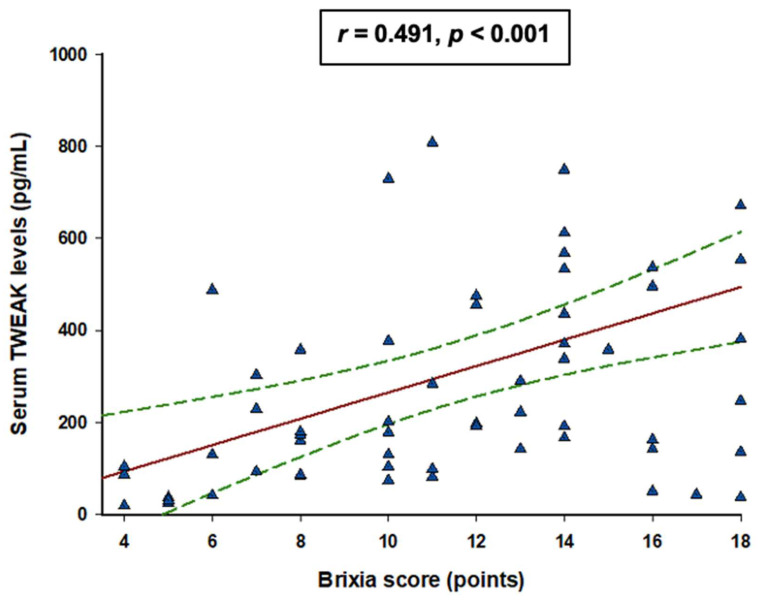
Spearman rank correlation analysis between Brixia score and serum TWEAK levels. The red line represents a correlation line, the dotted green lines represent 95% confidence interval, and blue triangles represent values from respective patients.

A significant positive correlation was found between Brixia score and serum levels of TWEAK (r = 0.491, *p* < 0.001) (Figure 2) Likewise, a positive correlation was found between serum levels of TWEAK at admission and CRP levels (r = 0.410, *p* = 0.002) as well as between TWEAK and hs-TnI levels (r = 0.463, *p* = 0.001). On the other hand, weak negative correlation was found between serum TWEAK at admission and testosterone levels (r = −0.310, *p* = 0.036). SAPS-II score, SOSIC scores, Horowitz index, TSH levels, and vitamin D levels did not correlate with TWEAK serum levels (Table 3).

**Figure 3:**
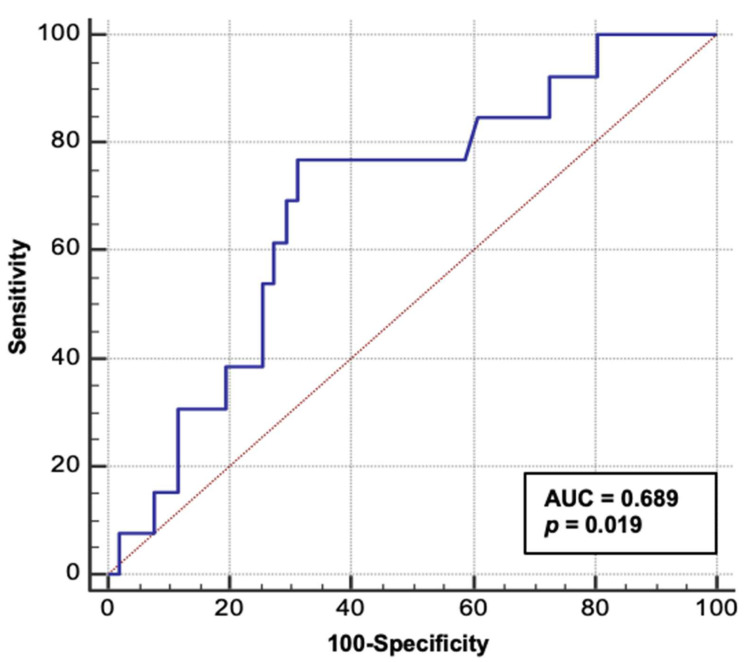
Receiver-operator characteristics (ROC) analysis of serum TWEAK at admission for in-hospital mortality. Abbreviations: AUC, area under the curve.

Receiver-operator characteristics analysis demonstrated that serum TWEAK at admission to ICU is a significant predictor of in-hospital mortality (AUC = 0.689, *p* = 0.019) (Figure 3).

## 4. Discussion

In the present study, for the first time, we showed that serum TWEAK upon admission to ICU in a severe form of COVID-19 may be a predictor of in-hospital mortality. Furthermore, we demonstrated that TWEAK levels continue to rise the first 7 days from admission to ICU, with subsequent reduction in values 14 days after admission to ICU but only in patients who survive until discharge. Finally, we established correlations between serum TWEAK and indices of both myocardial and lung injury as well as TWEAK and testosterone levels. To the best of our knowledge, this is the first study in which dynamic of serum TWEAK was analyzed in critically ill COVID-19 patients.

Association between TWEAK serum levels and in-hospital mortality is in line with the available data concerning the pathophysiological background underlying COVID-19 fatality. Namely, it has so far been well-established that inappropriate inflammatory response is a crucial factor in the development and progression of severe COVID-19 [27]. Upon entering the host cell, viral RNA triggers pattern recognition receptors (PRR), activating multiple mediators such as NFκβ and IRF3/7 that subsequently lead to the formation of Interferon I (IFNI) and secretion of multiple pro-inflammatory cytokines and chemokines [28,29]. Although in most cases, such response is coordinated by an array of regulatory pathways, in susceptible patients, the expected protective host immune response converts to harmful inflammation that further leads to end-organ damage and dampens the recovery from infection [30]. Interleukin-6 (IL-6) is one of the most widely explored cytokines in COVID-19. Multiple studies have demonstrated that IL-6 levels are elevated in patients who suffer from a severe form of COVID-19 [31,32,33]. Moreover, a study demonstrated that IL-6 and CRP in combination can predict the development of respiratory failure with substantial sensitivity and specificity [34]. In line with this, tocilizumab, an IL-6R inhibitor, has been associated with improved outcomes in critically ill COVID-19 patients [35,36,37]. As binding of TWEAK to Fn14 triggers downstream signaling that contributes to IL-6 secretion (among other pro-inflammatory cytokines), it is plausible that TWEAK-Fn14 axis might play a role in the early development of cytokine storm syndrome.

The principal mechanism by which TWEAK-Fn14 axis contributes to secretion of pro-inflammatory cytokines is activation of the NF-κB pathway, a central controlling unit of gene transcription during inflammation [38,39]. These inferences were established upon treatment of various cell types in vitro with TWEAK, which has been shown to induce the expression of known NF-κB target genes, such as B-cell lymphoma-2 (BCL-2), BCL-XL, matrix metalloproteinase-9 (MMP-9), and Fn14 itself [40,41,42]. Consistently, anti-TWEAK-neutralizing monoclonal antibodies were shown to reduce inflammatory cell infiltration in mice models of multiple sclerosis and rheumatoid arthritis, whereas Fn14-null mice demonstrated a deficient inflammatory response upon tissue injury [41,42,43,44,45]. Thus far, only one study addressed the role of serum TWEAK in patients with COVID-19 [46]. Kehribar et al. compared serum TWEAK levels between healthy controls and patients with COVID-19. The study showed that TWEAK levels were significantly higher in COVID-19 patients in comparison to controls. Interestingly, patients with COVID-19 with lung involvement, as demonstrated by computed tomography, had significantly higher TWEAK levels in contrast to counterparts with COVID-19 but with no lung involvement.

An observation that TWEAK levels return to baseline levels in patients who recover but not in those who die during in-hospital stay must be interpreted with caution. However, we may hypothesize that these levels reflect inflammatory state of patients. In patients who start to recover from severe COVID-19, inflammation begins to resolve, and therefore, the serum levels of pro-inflammatory biomarkers also fade. On the contrary, in patients who continue to deteriorate, multiorgan failure and nosocomial infections develop, thus sustaining the detrimental inflammatory response. Similar fluctuations of IL-6 serum levels were demonstrated in multiple populations [47,48,49,50,51]. For instance, Kergert et al. demonstrated that IL-6 levels at admission and 5 days after were relatively higher in patients who developed macrophage activation syndrome (MAS) or acute respiratory distress syndrome (ARDS) in comparison to the patients who did not [52].

Positive correlation between TWEAK and Brixia score, a semi-quantitative scoring system rating lung involvement, is consistent with the above-noted findings. Specifically, Brixia score correlates strongly with disease severity and outcomes and may even support the clinical decision making in patients with moderate-to-severe signs and symptoms of COVID-19 [53]. On a separate note, hs-TnI, a sensitive biomarker of myocardial injury, has been also shown to predict severity and mortality of COVID-19 infection [54,55,56,57].

Finally, we observed negative correlation between serum levels of testosterone and TWEAK. These results may be explained in light of the existing evidence. Low levels of serum testosterone have been associated with endothelial dysfunction and inflammation and may also predispose men to less-effective immune response against infectious agents, such as COVID-19. Notably, data suggest that male hypogonadism may trigger a detrimental cytokine dysfunction, including high circulating levels of IL-6, TNF-alpha, and IL-1beta, thus portending poor outcomes in critically ill COVID19 patients [58,59,60,61]. It is also important to address that pro-inflammatory cytokines further dampen release and action of testosterone, thus initiating a vicious cycle [62]. However, testosterone is a two-way street in COVID-19 [21]. On one hand, testosterone inhibits the release of pro-inflammatory cytokines, modulates the immune response, and attenuates oxidative stress and endothelial dysfunction. On the other hand, high testosterone levels might increase susceptibility and severity of COVID-19 through augmentation of TMPRSS2, which is crucial for cleaving and activation of SARS-CoV-2 spike protein during acute SARS-CoV-2 infection [63].

The present study bears several limitations. Firstly, the study was conducted exclusively on male population, thus rendering the conclusions on the female population impossible. The rationale for choosing male population only was to focus on the relationship between testosterone and both inflammation and outcomes. Secondly, this was a single-center study performed on a limited number of participants. Thirdly, all patients received standard-of-care corticosteroid treatment, which may have affected the serum values of TWEAK. Finally, a group of healthy controls and patients with mild-to-moderate COVID-19 were not included in the present study.

## 5. Conclusions

Despite the advent of variants that are highly infectious but cause less-severe disease, we must not neglect further research of this disease. As previously noted, one of the main reasons for high mortality during early COVID-19 pandemic was our lack of knowledge with regards to treatment of the disease. In addition, timely identification of patients in whom we expect clinical deterioration was also based on the knowledge from the pre-COVID-19 era. Therefore, it is now crucial to establish valid conclusions that will help us treat patients in future outbreaks of similar viruses. To accomplish this, the establishment of the pathophysiologic background is the most important aspect because the observed clinical data may lead to wrong inferences if we do not have this framework. Our results indicate that TWEAK may be one of the missing pieces in the puzzle of aberrant immune response in COVID-19. Specifically, the TWEAK/Fn14 axis has already been established as stimulator for secretion of IL-6, perhaps the most important mediator of cytokine storm syndrome, thus implying that TWEAK may have a role in pathogenesis of this syndrome. The fact that serum TWEAK levels rise in critically ill patients and then subsequently fall to baseline levels if clinical improvement occurs supports this hypothesis. Importantly, as anti-TWEAK therapy has proven useful in preclinical models of autoimmune diseases, and as lower serum levels of TWEAK are associated with better outcomes, it is possible that TWEAK may also represent a viable therapeutic target in COVID-19 and similar infections. Nevertheless, such conclusions still remain speculative. Finally, the observation that serum TWEAK negatively correlates with testosterone levels represents yet another evidence of complex, albeit undoubtedly negative, interaction between inflammation and reproductive hormone axis in COVID-19.

## Figures and Tables

**Table 1:** Characteristics of patients at admission to the RIC.

Variables	Death Event		Total (*n* = 66)	*p* *
	No (*n* = 53)	Yes (*n* = 13)		
Age (years)	54.2 ± 7.9	58.9 ± 6.0	55.1 ± 7.7	0.047
BMI (kg/m^2^)	28.6 ± 3.9	28.2 ± 2.7	28.5 ± 3.7	0.771
Disease duration at admission to hospital (days)	9.0 ± 2.6	8.6 ± 3.4	8.9 ± 2.8	0.690
Disease duration at admission to ICU (days)	11.1 ± 3.2	10.5 ± 5.3	11.0 ± 3.7	0.607
Duration of hospitalization (days)	16 (14–20)	23 (15–35)	17 (14–23)	0.199
ICU length of stay (days)	11.5 ± 6.7	20.3 ± 8.5	13.2 ± 7.9	<0.001
Duration of mechanical ventilation (days)	7.0 ± 4.8	19.2 ± 8.6	9.4 ± 7.5	<0.001
Nosocomial infection (n, %)	18 (34%)	10 (77%)	28 (42%)	<0.001
Fully vaccinated (n, %)	10 (19%)	2 (15%)	12 (18%)	0.772
Comorbidities				
Active smoking (n, %)	4 (7.6%)	1 (7.7%)	5 (7.6%)	0.852
Arterial hypertension (n, %)	14 (26.4%)	5 (38.5%)	19 (28.8%)	0.004
Diabetes mellitus (n, %)	2 (3.8%)	2 (15.4%)	4 (6.1%)	<0.001
Dyslipidemia (n, %)	33 (63%)	12 (54%)	45 (68%)	0.667
Hypothyroidism (n, %)	2 (3.8%)	1 (7.8%)	3 (4.5%)	N/A
Laboratory parameters				
SaO_2_ (%)	93.0 (90.4–96)	94.0 (86.6–96.0)	91.9 (90.3–96.0)	0.472
pH (units)	7.35 ± 0.07	7.34 ± 0.06	7.35 ± 0.07	0.475
pO_2_ (kPa)	5.8 ± 1.1	6.0 ± 0.7	5.8 ± 1.0	0.507
pCO_2_ (kPa)	9.8 ± 2.6	9.6 ± 2.1	9.8 ± 2.5	0.858
HCO_3_^−^ (mmol/L)	25.9 ± 2.4	25.5 ± 2.1	25.8 ± 2.3	0.629
Hemoglobin (g/L)	133.9 ± 12.0	131.9 ± 12.3	133.5 ± 12.0	0.591
Platelets (×10^9^/L)	264.1 ± 84.6	217.3 ± 87.1	254.9 ± 86.7	0.081
WBC (×10^9^/L)	10.0 ± 3.4	9.1 ± 4.6	9.8 ± 3.6	0.413
Neutrophiles (%)	88.9 ± 3.9	87.2 ± 5.0	88.6 ± 4.2	0.161
Lymphocytes (%)	6.8 ± 2.9	8.7 ± 4.4	7.2 ± 3.3	0.070
Monocytes (%)	3.51 ± 1.76	3.69 ± 1.72	3.5 ± 1.7	0.735
Eosinophiles (%)	0.37 ± 0.24	0.35 ± 0.22	0.36 ± 0.23	0.832
CRP (mmol/L)	87.2 ± 66.9	84.9 ± 35.2	86.5 ± 61.7	0.868
LDH (umol/L)	445.8 ± 221.8	605.1 ± 225.9	477.2 ± 222.9	0.024
D-dimers (mg/L)	2.17 (1.51–4.01)	3.08 (1.77–6.65)	2.34 (1.63–4.19)	0.305
Fibrinogen (g/L)	6.7 ± 1.3	6.5 ± 1.3	6.6 ± 1.4	0.507
INR	0.96 ± 0.06	0.99 ± 0.11	0.96 ± 0.07	0.199
aPTT (s)	22.2 ± 3.4	22.6 ± 3.2	22.3 ± 3.3	0.647
hsTnI (ng/L)	9.1 (5.9–13.7)	18.2 (9.9–32.8)	9.9 (6.2–16.3)	0.002
Blood glucose (mmol/L)	9.5 ± 2.6	10.8 ± 5.2	9.8 ± 3.3	0.206
Lactate (mmol/L)	1.5 (1.1–2.0)	1.4 (1.08–1.55)	1.5 (1.1–2.0)	0.235
Urea (mmol/L)	7.9 ± 2.1	8.8 ± 4.4	8.1 ± 2.7	0.289
Creatinine (mmol/L)	75.6 ± 15.4	84.2 ± 28.4	77.3 ± 18.7	0.142
Testosterone (nmol/L)	0.74 (0.44–1.49)	0.85 (0.60–1.50)	0.77 (0.44–1.49)	0.723
TSH (mIU/L)	0.34 (0.19–0.64)	0.25 (0.17–0.62)	(0.18–0.63)	0.699
fT3 (pmol/L)	2.37 ± 0.42	2.53 ± 0.90	2.41 ± 0.54	0.353
fT4 (pmol/L)	15.69 ± 3.55	12.04 ± 3.11	15.52 3.33	0.478
Vitamin D (nmol/L)	40.0 ± 18.6	27.4 ± 17.0	37.7 ± 18.8	0.040

Data are expressed as mean ± SD, number (percent), or median (interquartile range). * Mann–Whitney U test, chi-square test, or Student’s *t*-test. Abbreviations: CRP, C-reactive protein; HCO_3_^−^, bicarbonate; LDH, lactate dehydrogenase; pO_2_, partial pressure of oxygen in the blood; pCO_2_, partial pressure of carbon dioxide in the blood; SpO_2_, oxygen saturation; WBC, white blood cells; hs-TnI, high-sensitivity troponin I; RIC: Respiratory Intensivist Center; ICU: intensive care unit; TSH: thyroid stimulating hormone; BMI: Body mass index; INR: International normalized ratio; aPTT: Activated Partial Thromboplastin Time.

**Table 2:** Clinical scores of interest.

Variables	Death Event		Total (*n* = 66)	*p*
	No (*n* = 53)	Yes (*n* = 13)		
Horowitz index	91.1 ± 30.9	84.1 ± 24.9	89.7 ± 29.7	0.449 †
SAPS II	29 (27–32.5)	32 (29–34)	29 (27–34)	0.168 *
SOSIC-1	27.6 (21.1–36.0)	35.4 (31.7–41.9)	30.3 (22.9–36.4)	0.013 *
SOSIC-7	6.4 (3.2–25.8)	38.5 (31.1–43.4)	12.9 (3.9–33.1)	<0.001 *
SOSIC-14	4.4 (2.0–11.9)	48.9 (46.0–55.4)	27.6 (2.8–48.0)	<0.001 *
Brixia score	11.5 ± 3.8	14.9 ± 2.7	12.2 ± 3.9	0.004 †

Data are expressed as mean ± SD, number (percent), or median (interquartile range). * Mann–Whitney U test; † Student’s *t*-test. Abbreviations: SOSIC, Survival of Severely Ill COVID; SAPS II, Simplified Acute Physiology Score.

**Table 3:** Correlation between serum TWEAK levels at admission to ICU and variables of interest.

Variable	r *	*p*
CRP (mmol/L)	0.410	0.002
hs-TnI (ng/L)	0.463	0.001
Testosterone (nmol/L)	−0.310	0.036
SAPS-II	0.233	0.064
SOSIC-1	−0.072	0.574
SOSIC-7	0.115	0.387
SOSIC-14	0.440	0.054
TSH (mIU/L)	−0.125	0.326
Vitamin D (nmol/L)	−0.012	0.928
Horowitz index	−0.122	0.335

* Spearman rank correlation coefficient. Abbreviations: CRP, c-reactive protein; hs-TnI, high-sensitivity troponin I; SOSIC, Survival of Severely Ill COVID; SAPS II, Simplified Acute Physiology Score; TSH, thyroid-stimulating hormone.

## Data Availability

The data presented in this study are available on request from the corresponding author. The data are not publicly available because some of the data set will be used for further research.
